# Case Report: Successful multimodal management of FIRES in a pediatric patient using anakinra, ketogenic diet, and induced hypothermia

**DOI:** 10.3389/fped.2025.1621719

**Published:** 2025-11-10

**Authors:** Mariagiovanna Caporale, Giulia Lais, Antonio Gulli’, Carla Caporale, Marco Piastra, Giorgio Conti

**Affiliations:** 1 Department of Anesthesiology and Intensive Care Medicine, Catholic University of The Sacred Heart, Fondazione “Policlinico Universitario A. Gemelli” IRCCS, Rome, Italy; 2 Department of Woman and Child Health and Public Health, Child Health Area, Catholic University of the Sacred Heart, Rome, Italy

**Keywords:** refractory epilepsy, febrile illness-related epilepsy syndrome, anakinra, induced hypothermia, ketogenic diet, new onset refractory status epilepticus, antiepileptic drugs

## Abstract

Febrile infection–related epilepsy syndrome is a devastating, refractory epileptic encephalopathy that typically occurs in school-aged, otherwise healthy, children after a brief, non-specific, febrile illness. In this study, we discuss a case of a previously healthy 10-year-old girl with FIRES in whom high dosages of conventional and non-conventional antiepileptic drugs were ineffective in treating refractory status epilepticus, after which a treatment involving intravenous corticosteroids, immunoglobulins, a ketogenic diet, and induced hypothermia was adopted. We conducted a diagnostic workup that included lumbar puncture, blood tests, continuous electroencephalogram monitoring, brain MRI, and autoimmunity and infectious disease panels to shed light on the etiology of the condition. Our patient responded to immunosuppressive therapy and the ketogenic diet, and her condition gradually improved to full recovery with only mild neurocognitive sequelae. Although patients with FIRES have been described in the literature, optimal management has not yet seen the light of day, and therefore, prospective cohort studies are needed. This case highlights the potential benefit of a timely, multimodal therapeutic approach combining immunomodulation, metabolic support, and neuroprotection. Early administration of anakinra and initiation of a ketogenic diet may help control seizures and reduce the duration of ICU stay. Prompt diagnosis and interdisciplinary care are essential to improve outcomes in patients with this life-threatening pediatric condition.

## Highlights

The objective of this study is to highlight the importance of timely identification of FIRES when children or young adults suffer from overwhelming seizures or status epilepticus, although no previous medical history of epilepsy has been recorded in them.

Prompt treatment of refractory status epilepticus is necessary to limit poor neurological outcome.
Early initiation of immunosuppressive therapy may reduce dependence from mechanical ventilation and ICU-related complications.

## Introduction

Febrile infection–related epilepsy syndrome (FIRES) is a devastating, refractory epileptic encephalopathy that typically occurs in school-aged, otherwise healthy, children after a brief, non-specific, febrile illness. Seizures appear 24 h to 2 weeks after a febrile episode and are usually focal seizures; however, these seizures might evolve into bilateral and multifocal types with autonomic features and a strong tendency to progress into a prolonged super-refractory status epilepticus (SRSE), which may last months and can be poorly responsive to common medications (1). FIRES is considered a subcategory of New Onset Refractory Status Epilepticus (NORSE). Although it is a rare condition, it should be taken into consideration when evaluating children with resistant status epilepticus (SE). Unfortunately, 1 in 10 children dies from status epilepticus or intensive care complications; moreover, nearly all survivors have chronic epilepsy requiring medical management and develop cognitive impairment related to the age at which FIRES presented and the duration of burst-suppression (BS) coma (2).

## Case presentation

We present a case of an otherwise healthy 10-year-old girl with a recent febrile status, who was referred to our Pediatric Intensive Care Unit (PICU) from a second-level hospital because of recurrent drug-resistant focal motor seizures. The patient experienced non-specific symptoms (cacosmia, dysphagia, and drowsiness) a couple of days before hospital admission and had a mild pharyngitis one month prior to admission; the parents also reported a tick bite. The family pediatrician started the patient on empiric antibiotic therapy. After 5 days of a mild febrile illness, the patient was admitted to the emergency department because of a generalized tonic–clonic seizure. After a second episode of focal seizure, characterized by confusion and gaze deviation, the patient was put on midazolam (MDZ), with transient improvement in consciousness. Diagnostic workup that included blood tests and a computed tomography (CT) scan was unremarkable with no clear infectious etiology determined. Magnetic resonance imaging (MRI) showed “a 5 mm area of signal alteration, characterized by a reduction of the restricted diffusivity in the diffusion weighted imaging (DWI) sequences, as for cytotoxic edema, compatible with reversible lesion of the splenium of the corpus callosum.” Despite an intravenous administration of benzodiazepine (BDZ), the seizures increased in frequency and length of duration, leading to gas exchange impairment; thus, the patient was transferred to our PICU.

**Figure 1:**
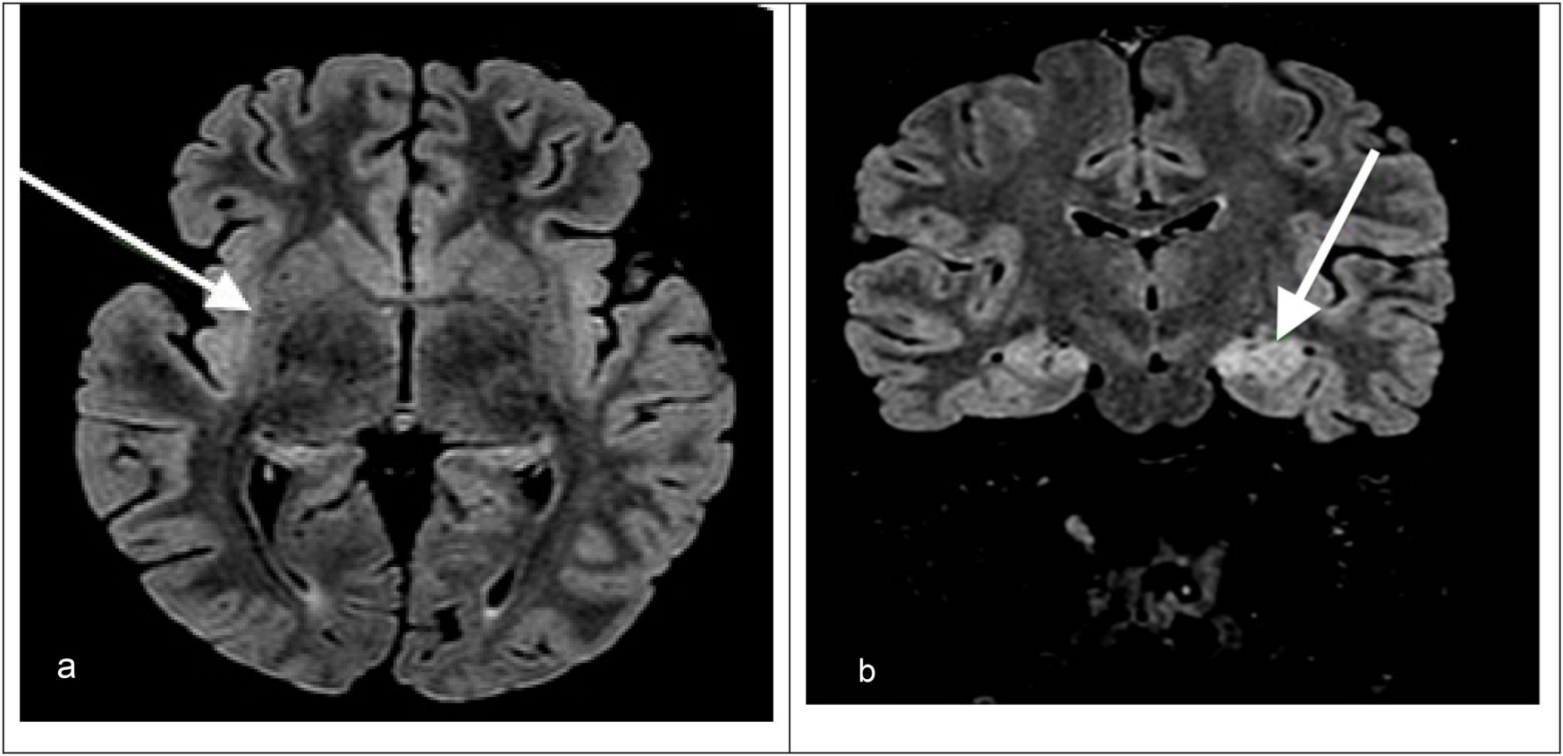
(**a**) Axial T2-FLAIR MRI image showing hyperintensity in the right hippocampus and right claustrum (arrow). (**b**) Coronal T2-FLAIR MRI image showing bilateral hippocampal hyperintensities, which are more prominent on the left. The arrow indicates left hippocampal involvement.

Upon admission to our hospital, a comprehensive examination was conducted, which included blood microbiological tests, metabolic and cerebrospinal fluid (CSF) analyses (including autoimmunity), and continuous electroencephalogram (cEEG) monitoring. he patient's condition gradually deteriorated, necessitating invasive ventilatory support. Continuous EEG monitoring showed an unstructured background tracing characterized by global slow large-amplitude activity. The monitoring registered almost continuous autonomic focal seizures with right EEG changes and subsequent generalization. Therapy was therefore initiated, which required the use of propofol, ketamine, phenytoin, and phenobarbital. After a second lumbar puncture (LP), intravenous steroids and immunoglobulins were also administered. A transitory reduction of seizure frequency was obtained only with high doses of the abovementioned antiepileptic drugs; however, cEEG recorded transitory BS patterns, with both right and left focal motor seizures and secondarily generalized seizures fulfilling the criteria for refractory SE. We decided to repeat an MRI, which showed hyperintensity signal alteration in the T2 FLAIR involving the hippocampus bilaterally (left > right) with concomitant alteration at the level of the claustrum (Figure 1).

Based on the seizure type and frequency, ranging from 20 to 35 per hour, as well as the EEG, MRI, and clinical features, a diagnosis of FIRES was made. We decided to continue antiepileptic therapy with propofol, MDZ, lacosamide, levetiracetam, and phenobarbital and integrate it with a ketogenic diet. In the meantime, we obtained the results of the extensive analysis performed on the CSF, which were consistent with those obtained for the inflammation of the central nervous system (CNS), while the result of the autoimmunity panel was negative. Despite maximal antiepileptic therapy, cEEG showed subcontinuous critical episodes, after which 72 h of therapeutic hypothermia (temperature 33.5°C) was initiated with anakinra. After 48 h of the induced hypothermia, the patient became febrile, following which a full microbiological panel was drawn. The subsequent day, we started rewarming the patient; however, due to the persistence of respiratory failure and ventilator dependence, the patient underwent surgical tracheostomy. The patient developed ventilator-associated pneumonia (VAP) on day 21 of the PICU stay and required antibiotic therapy.

The patient’s neurological condition gradually improved, after which we started sedative deescalation, which also enabled successful weaning from mechanical ventilation. After 1 week, the patient was awake, came into contact with the external environment, and was able to execute simple tasks. She was discharged from the PICU after 32 days with a Glasgow Coma Scale of 11 (E4, M6, Vt) and remained in the pediatric neuropsychiatry ward for two months. EEG tracings are not given here because there was continuous bedside video-EEG monitoring in the PICU, which did not allow export of the recorded data.

## Investigations

FIRES typically begins with a mild non-specific febrile illness; 24 h to 2 weeks later, seizures start presenting along with an increase in frequency and intensity that quickly escalates into status epilepticus. For the patient in this study, a brain CT scan was performed upon first hospital admission to exclude the presence of organic lesions, and an EEG was recorded for crisis characterization. Routine laboratory tests were conducted, including a complete blood count, blood chemistry with electrolytes, a coagulation assay, and inflammatory markers, which showed no significant alterations. Because of the refractoriness of the crisis despite BDZ therapy and the need for oxygen supplementation, the patient underwent an urgent brain MRI that showed a 5-mm area of cytotoxic edema compatible with a reversible lesion of the corpus callosum.

From a microbiological point of view, plasma serology was performed. It demonstrated IgM positivity for herpes simplex virus and cytomegalovirus and IgM and IgG positivity for SARS-CoV-2, while it showed negative results for adenovirus, varicella zoster virus, Epstein–Barr virus, coxsackievirus, influenza virus, parvovirus B19, and mycoplasma pneumoniae. LP was performed, and a CSF analysis showed elevated levels of glucose and protein and the absence of cells. The result of the molecular film array test was positive for *Streptococcus agalactiae*. Samples of blood and CSF were also collected for conducting more specific tests—including molecular assays for Tuscan virus, West Nile virus, Usutu virus, *Borrelia*, and *Rickettsia*, as well as serological tests for antibody detection—and all of the tests ultimately yielded negative results.

Other blood and CSF samples were collected for an extensive autoimmune evaluation that included an evaluation for oligoclonal bands (that were absent) and the following antibodies: antiglutamate receptor, antionconeural antigen, antiaquaporin, anti-myelin oligodendrocyte glycoprotein (anti-MOG), anti-glutamic acid decarboxylase (anti-GAD), antigliadin, antitransglutaminase, antiendomysium, anti-neutrophil cytoplasmic antibodies (anti-ANCA), anti-deoxyribonucleic acid (anti-DNA), anti-extractable nuclear antigen (anti-ENA), antimitochondrial, and antinucleus. The evaluation results were all negative, and complement concentrations were all within range. Only one positive result was obtained for the lupus anticoagulant.

Serial measurements of cytokine levels were ascertained in CSF samples during the initiation of therapy with anakinra. The initial values showed an increase in the concentration of IL-1 by 50%, while IL-2 and IL-6 increased three times compared with the normal levels; on the other hand, TNF was reduced. These findings were compatible with those observed in inflammatory activation of the CNS. Cytokine reduction was observed in subsequent measurements after the initiation of immunomodulator therapy.

Electrodes for cEEG monitoring were placed as soon as the child was transferred into the PICU. As the clinical condition worsened and the crisis transformed into refractory status epilepticus, there was a progressive slowing of theta and delta rhythms present bilaterally in the frontotemporal region, with right-hemisphere predominance and phases of BS. Numerous crises were recorded in concomitance with attempts at sedative reduction, mainly starting from the right hemisphere with rapid global diffusion; other epileptiform abnormalities, including sharp waves and spikes, were frequently observed during interictal periods.

MRI was repeated once during the PICU stay, showing non-specific signal alteration in the left hippocampus and left claustrum without signs of blood–brain barrier (BBB) disruption and resolution of the edema at the level of the corpus callosum, which was compatible with the hypothesis of an autoimmune condition (Figure 1).

## Differential diagnosis

The etiology of FIRES may be related to an inflammatory or autoimmune mechanism, but its exact pathophysiology remains unknown, and there is no specific test available, making a clinical diagnosis possible only after the exclusion of other potentially treatable causes of SRSE (1). The initial stabilization of the patient should aim at interrupting the critical electrical activity with eventual achievement of BS when necessary to protect brain function, with a frequent need for advanced airway control. Empirical antibiotic and antiviral treatment should be initiated promptly upon suspicion of CNS infection, even before diagnostic confirmation. A diagnostic workup should follow in tandem with diagnostic confirmation. Immunosuppressive therapy, to target the inflammatory phenomenon behind the clinical manifestation, should be started only after the active infection has been reasonably excluded. In our patient, the clinical features—characterized by the absence of neck rigidity and fever, normal white blood cell (WBC) count, normal C-reactive protein and procalcitonin levels, and clear CSF analysis showing only mildly elevated protein levels (with no decrease in glucose and no WBC)—made an infectious disease etiology unlikely despite *S. agalactiae* positivity upon molecular testing, indicating an autoimmune process as the most likely cause. The isolated positive result for the lupus anticoagulant was interpreted as a transient finding, commonly observed during systemic inflammation or infections, and not associated with clinical or laboratory features suggestive of antiphospholipid syndrome.

## Treatment

Empirical antibiotic therapy for meningitis was started upon the patient's admission to the PICU, with ceftriaxone 2 g twice a day, ampicillin 3 g four times a day, and acyclovir 400 mg three times per day as soon as the first lumbar puncture was performed (within 2 h of PICU admission).

Because of the progressive worsening of the child's clinical conditions with frequent incoming focal crises associated with episodes of desaturation, it became necessary to provide ventilatory support with continuous positive airway pressure (CPAP) and sedation with midazolam (up to 0.01 mg/kg/min), dexmedetomidine (up to 1.4 μg/kg/h), fentanyl (up to 0.033 μg/kg/min), and an increasing dose of propofol (up to 0.125 mg/kg/min) to attain BS. Consciousness impairment caused by the crisis and the need for sedatives led to advanced airway control, first through endotracheal intubation and subsequently through surgical tracheostomy, to facilitate weaning from both drugs and invasive ventilation.

Anticonvulsive therapy was implemented with the progressive addition of levetiracetam (500 mg TID, approximately 37.5 mg/kg/day for a 40-kg child), lacosamide (50 mg × 2, the 100 + 50 mg), phenobarbital (bolus 100 mg–2.5 mg/kg), phenytoin (bolus 600 mg–15 mg/kg, then 200 mg  ×3 added to therapy), ketamine (continuous infusion of 0.01 mg/kg/min), and cannabidiol (up to 300 mg × 2 per os), all of which proved to be extremely useful in the recovery phase by facilitating weaning from intravenous drugs.

After excluding the presence of active bacterial meningitis, corticosteroid therapy was started with methylprednisolone 20 mg/kg/die (800 mg/day for 5 days) and intravenous immunoglobulins (IVIG) (two 48-h cycles, using a 1-g/kg dose for each cycle).

Lack of improvement in the patient’s condition led to an escalation of therapy. First, the patient underwent therapeutic hypothermia through the use of a cooling mattress with target therapy 33.5 °C for 72 h, followed by slow rewarming (0.5 °C/h). The clinical picture was further complicated by a concomitant VAP causing fever, increased need for ventilatory support, and evidence of lung consolidation on radiological imaging, which was first treated with cefepime and linezolid, and then treated with amoxicillin-clavulanate and linezolid upon isolation of methicillin-sensitive *Staphylococcus aureus* (MSSA) in a tracheal aspirate sample.

Then, biological therapy with anakinra was administered with a starting dose of 5 mg/kg (100 mg subcutaneously twice daily), which was soon after doubled to 200 mg subcutaneously twice a day. Anakinra is a recombinant and slightly modified version of the human IL-1 receptor antagonist protein, whose therapeutic role has been described in numerous autoimmune conditions. LP was repeated during therapy to monitor the reduction of CSF cytokine levels (3, 4).

Simultaneously, enteral nutrition was switched to a ketogenic formulation (4:1) high in fat and low in carbohydrate, with sufficient protein to mimic the fasting state. This was administered through the nasogastric tube, and the presence of ketone bodies in the urine was monitored daily.

## Evolution, outcome, and follow-up

After twenty days of high-dose sedation on invasive mechanical ventilation, the patient began to recover. Weaning from both intravenous sedation and ventilatory support was particularly challenging. Relapse occurred, characterized by a few episodes of buccal automatisms and loss of contact with the environment; however, it was no longer necessary to maintain the state of BS due to a progressive improvement of EEG activity.

The child was transferred to the pediatric neuropsychiatric ward after 32 days in the PICU, conscious and awake, able to follow simple commands, and breathing spontaneously through tracheostomy with minimal oxygen supplement. She continued to be fed a ketogenic diet for a few weeks and underwent a progressive switch to a more varied diet. She underwent rehabilitation, regained speech, and eventually achieved tracheostomy closure. Her MRI showed stable results with the persistence of bilateral signal alteration at the level of the claustrum and hippocampus. Brain electrical activity was subsequently recorded for 48 h during the patient's stay in the ward and showed the presence of an abnormally rapid rhythm during the state of arousal and two brief focal crises starting from the right frontotemporal region with later bilateral spread. During her stay in the ward, the patient started to note pain associated with sensory and motor alterations of the inferior right limb with evidence of the right sciatic nerve's axonal damage on electromyography. The MRI showed evidence of asymmetric neuropathy of the right sciatic nerve, with acute denervation of the right quadriceps femoris muscle for which therapy with gabapentin was started.

At follow-up, the patient presented slightly below-average cognitive development, with an IQ of 78. EEG showed altered cerebral electrical activity with a disorganized background and paroxysmal discharges over the bilateral fronto-centro-temporal regions. The patient continues to experience occasional focal aware seizures, mainly characterized by oral automatisms and behavioral arrest. She is currently being treated with clobazam, cannabidiol, and lacosamide and remains under neurological follow-up. Despite this, the patient has returned to school and is being provided learning support.

## Discussion

**Figure 2:**
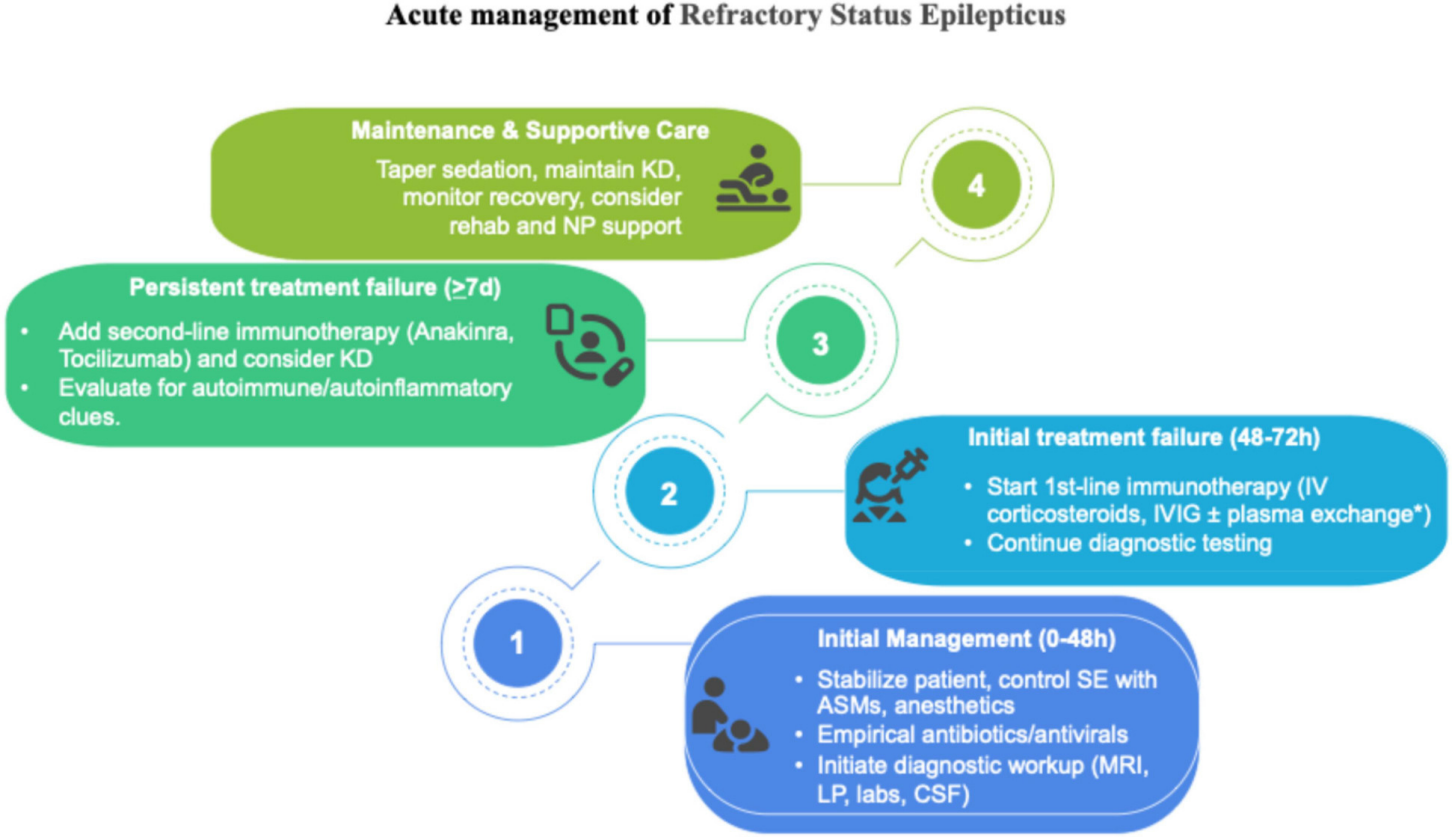
Pediatric treatment algorithm adapted from Wickstrom et al. (8). *There is no recommendation concerning plasma exchange; it may be used, but should not delay subsequent steps.

MRI findings such as hyperintensities in the claustrum and hippocampi, particularly when bilateral, have been described in FIRES and may reflect seizure-induced or inflammatory injury (Figure 1). In particular, bilateral hippocampal and claustrum involvement has been associated with prolonged status epilepticus and may indicate underlying neuroinflammation (5). Although FIRES is a rare syndrome, several hundred cases have been reported in the literature. For example, the German FIRES registry alone includes 93 children (6), and multiple case series have been reported from Europe, Asia, and the Americas, with cohorts ranging from 12 to over 70 patients (4, 5, 7). The pathophysiology of FIRES is poorly understood. However, some evidence suggests an inflammatory condition involving the central nervous system, characterized by an increase in the levels of cytokines and chemokines in the CSF that exceed those observed in plasma (1, 2). To date, almost all cases reported in the literature describe an infectious episode with a non-specific influenza-like illness prior to SE onset; thus, FIRES may be considered a postinfectious immune system dysregulation that has a predilection for the central nervous system in young and healthy, yet vulnerable, individuals (2, 4). Preliminary evidence suggests that immunosuppressive treatments such as high-dose corticosteroids, intravenous immunoglobulins, plasma exchanges, and the more novel anakinra, a recombinant antagonist of IL-1 receptor, might be helpful, but their efficacy has not been confirmed (1, 2). Plasmapheresis was not performed in our patient because, at the time of clinical decision-making, the patient's EEG and imaging features were suggestive of an autoinflammatory rather than an autoimmune encephalitic process (i.e., typical of FIRES), and autoantibody results were still pending. Given the lack of specific evidence of an autoimmune cause, and based on current international recommendations (8), we prioritized the use of immunomodulatory therapy with corticosteroids, IVIG, anakinra, and a ketogenic diet. A summary of the decisional algorithm of refractory status epilepticus is illustrated in Figure 2. As the patient showed gradual clinical improvement following this multimodal approach, we did not escalate to plasmapheresis. Plasmapheresis could still be considered an option within the first 72 h, but it should not delay subsequent steps.

The role of IL-1 in epilepsy has been demonstrated in animal models. A genetic analysis of 19 children with FIRES found a significant association with RN2 allele repeats of the IL1RN, which correlate with higher levels of IL-1β and lower levels of IL-1Ra, which could lead to an unopposed pathologic inflammatory state (2, 9). Even though only a few studies have analyzed CSF proinflammatory cytokine levels in relation to seizures, we have observed a correlation between a reduction in trend and clinical improvement with treatment over time. Mild hypothermia at 33 °C is believed to reduce proinflammatory cytokine levels and protect the integrity of the BBB that is disrupted by the neuroinflammatory state (1). Thus, neuroprotection is the rationale for the therapeutic use of hypothermia in the treatment of FIRES. A ketogenic diet is characterized by a low carbohydrate and high fat and protein intake that mimics the fasting state and exhibits anti-inflammatory properties in animal models (1, 2). The β-hydroxybutyrate generated inhibits the proteolytic activity of caspase-1, thereby reducing the release of IL-1β; its benefit in FIRES has been suggested by several small case series describing seizure resolution after a median of one week from diet initiation, matching what we observed in our patient (1, 2). Despite slow and progressive recovery and the development of refractory epilepsy, our patient was discharged with only mild cognitive impairment and minor residual EEG abnormalities. The prognosis of FIRES is extremely poor with a mortality rate of 9%–18% in the acute phase and severe neurological sequelae persisting in a majority of the survivors (48% have severe intellectual disabilities or remain in a vegetative state, 26% have mild to moderate intellectual disabilities and learning disabilities, 26% retain a normal intelligence quotient, and 87% exhibit residual or refractory epilepsy) (2). The aim of our study was to raise awareness and promote prompt therapy initiation. Prospective studies are needed to confirm therapy efficacy and investigate further options, such as the use of intrathecal dexamethasone and tacrolimus, a humanized antibody against IL-6 receptors, which is associated with a decrease in the IL-6 CSF concentration, and the use of inhalational anesthetic agents as salvage therapy.

## Summary

**Figure 3:**
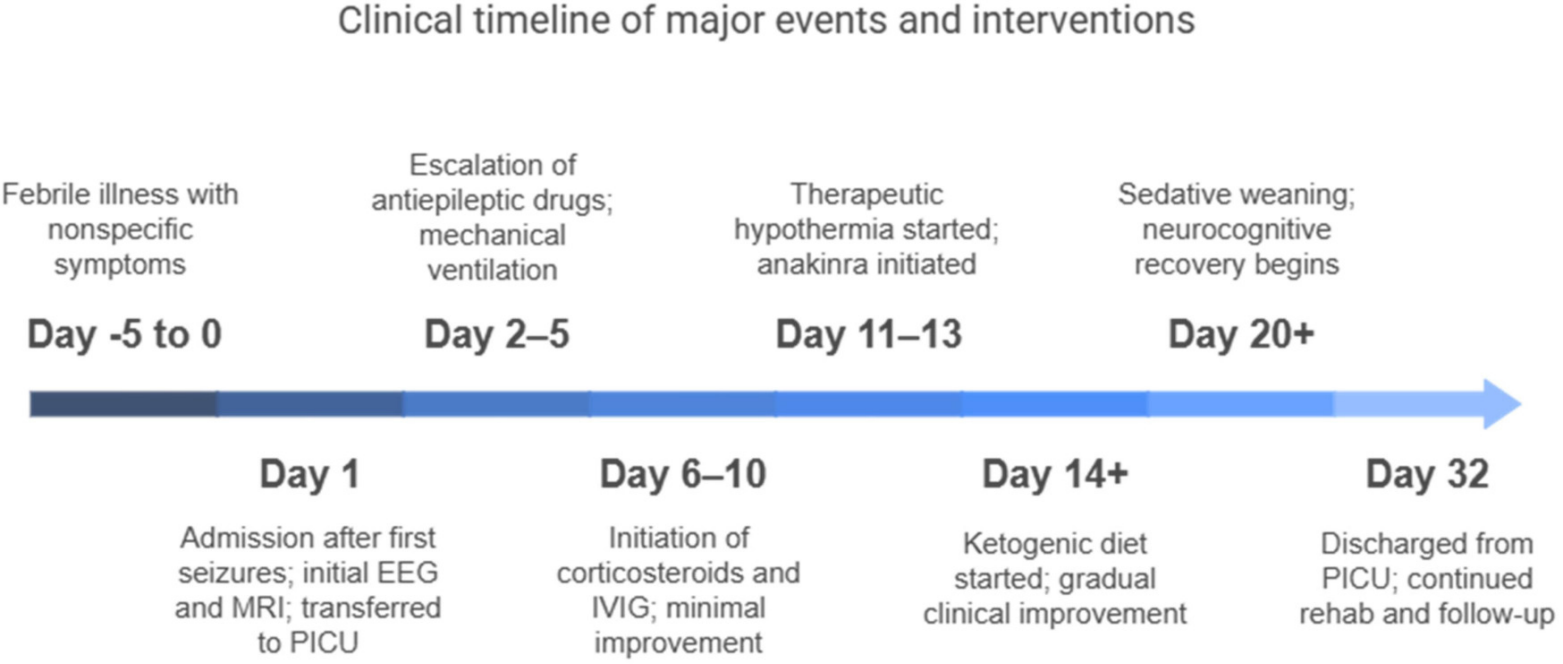
Clinical timeline of major events and interventions.

FIRES is a rare epileptic encephalopathy that primarily affects children without a prior history of epilepsy and is characterized by a febrile infection two weeks to 24 h before the onset of status epilepticus (1). The exact pathophysiology is unknown, but evidence suggests an autoinflammatory-mediated process. In this study, we reported a case of a 10-year-old girl with an unremarkable medical history who was admitted to our PICU with relapsing focal and secondarily generalized seizures after three days of a febrile illness. We conducted a workup that included continuous EEG monitoring, blood and CSF tests, autoimmunity and infectious disease panels, and brain MRI. Our initial treatment utilized conventional antiepileptic drugs. A diagnosis via clinical exclusion was made of FIRES, supported by compatible patterns of EEG and MRI. Initial empiric antibiotic therapy was started, and only after the infectious nature of the disease was ruled out, high-dose steroids and immunoglobulins were administered, achieving only minimal clinical or electrical improvement. After 72 h of moderate hypothermia, therapy was escalated to include the human recombinant immunomodulator anakinra and a ketogenic diet following recommendations from the literature (1, 2). The EEG patterns and clinical conditions gradually improved, allowing the child to be discharged with only minor neurocognitive sequelae. A summary of our case report is illustrated in Figure 3. Despite being a rare condition, clinicians should be aware of FIRES when managing children or young adults with refractory seizures because prompt diagnosis and treatment may be of utmost importance for limiting ICU-related complications, such as mechanical ventilation dependence and infections. In particular, early anakinra initiation has been associated with a shorter duration of mechanical ventilation, ICU stay, and overall hospital stay (4). The duration of burst-suppression coma, which may be necessary at times, also seems to have an impact on the severity of cognitive impairment.

## Take-home messages

FIRES is a condition of refractory status epilepticus that may present in children or young adults after a febrile illness.
The pathogenesis of FIRES is poorly understood, but proinflammatory cytokines seem to play a key role.
Immunosuppressive therapy with steroids, immunoglobulins, anakinra, and induced hypothermia may help reverse status epilepticus.
A ketogenic diet probably contributes to the resolution of seizures and a good outcome.
Interdisciplinary support is essential to optimize the management of patients with FIRES during both the acute phase and the long-term recovery phase.

## Patient perspective

The patient's family expressed deep appreciation for the multidisciplinary approach and the dedication shown by the care team. They reported being actively involved in decision-making and were relieved to witness the patient's cognitive recovery despite the complexity and duration of intensive care.

## Data Availability

The original contributions presented in the study are included in the article/Supplementary Material, and further inquiries can be directed to the corresponding author.
